# Cerebral blood flow in striatum is increased by partial dopamine agonism in initially antipsychotic-naïve patients with psychosis

**DOI:** 10.1017/S0033291723000144

**Published:** 2023-10

**Authors:** Kirsten Borup Bojesen, Birte Yding Glenthøj, Anne Korning Sigvard, Karen Tangmose, Jayachandra Mitta Raghava, Bjørn Hylsebeck Ebdrup, Egill Rostrup

**Affiliations:** 1 Center for Neuropsychiatric Schizophrenia Research (CNSR) & Center for Clinical Intervention and Neuropsychiatric Schizophrenia Research (CINS), Mental Health Center Glostrup, University of Copenhagen, Glostrup, Denmark; 2 Department of Clinical Medicine, Faculty of Health and Medical Sciences, University of Copenhagen, Copenhagen, Denmark; 3 Functional Imaging Unit, Department of Clinical Physiology, Nuclear Medicine and PET, Rigshospitalet, Glostrup, Denmark

**Keywords:** Antipsychotic treatment, antipsychotic-naïve, aripiprazole, cerebral blood flow, first-episode psychosis, first-episode schizophrenia, partial dopamine agonist, perfusion

## Abstract

**Background:**

Resting cerebral blood flow (rCBF) in striatum and thalamus is increased in medicated patients with psychosis, but whether this is caused by treatment or illness pathology is unclear. Specifically, effects of partial dopamine agonism, sex, and clinical correlates on rCBF are sparsely investigated. We therefore assessed rCBF in antipsychotic-naïve psychosis patients before and after aripiprazole monotherapy and related findings to sex and symptom improvement.

**Methods:**

We assessed rCBF with the pseudo-Continuous Arterial Spin Labeling (PCASL) sequence in 49 first-episode patients (22.6 ± 5.2 years, 58% females) and 50 healthy controls (HCs) (22.3 ± 4.4 years, 63% females) at baseline and in 29 patients and 49 HCs after six weeks. RCBF in striatum and thalamus was estimated with a region-of-interest (ROI) approach. Psychopathology was assessed with the positive and negative syndrome scale.

**Results:**

Baseline rCBF in striatum and thalamus was not altered in the combined patient group compared with HCs, but female patients had lower striatal rCBF compared with male patients (*p* = 0.009). Treatment with a partial dopamine agonist increased rCBF significantly in striatum (*p* = 0.006) in the whole patient group, but not significantly in thalamus. Baseline rCBF in nucleus accumbens was negatively associated with improvement in positive symptoms (*p* = 0.046), but baseline perfusion in whole striatum and thalamus was not related to treatment outcome.

**Conclusions:**

The findings suggest that striatal perfusion is increased by partial dopamine agonism and decreased in female patients prior to first treatment. This underlines the importance of treatment effects and sex differences when investigating the neurobiology of psychosis.

## Introduction

Adequate resting cerebral blood flow (rCBF) is essential for healthy brain function, but rCBF is altered in medicated patients with psychotic disorders (Goozee, Handley, Kempton, & Dazzan, 2014; Pollak et al., 2018). However, the cause and clinical impact of the rCBF alterations are sparsely investigated in patients with psychosis. RCBF alterations may reflect illness pathophysiology or an effect of antipsychotic treatment (Goozee et al., 2014). Studies of twins and antipsychotic-naïve patients with psychotic disorder can provide insight into rCBF alterations in the illness pathophysiology, whereas studies of patients and healthy controls (HCs) before and after antipsychotic treatment or challenge studies may illuminate the effect of antipsychotics. Support for rCBF alterations in psychosis pathophysiology comes from studies of subjects at ultra-high risk for psychosis (UHR) and subclinical psychotic-like experiences (Allen et al., 2016, 2018; Modinos et al., 2018), and a recent twin study that found a relation between increased striatal and thalamic rCBF and schizophrenia spectrum disorder (Legind et al., 2019). However, an effect of antipsychotic treatment cannot be ruled out in the twin study as patients were in the chronic phase of illness (Legind et al., 2019). Studies of antipsychotic-naïve patients can overcome the confounding effect of antipsychotic treatment. The majority of existing rCBF studies of antipsychotic-naïve patients do not find abnormal perfusion in striatal areas (Andreasen et al., 1997; Catafau et al., 1994; Corson, O'Leary, Miller, & Andreasen, 2002; Parellada et al., 1994; Scottish Schizophrenia Research Group, 1998), although reduced (Vita et al., 1995) and increased (Early, Reiman, Raichle, & Spitznagel, 1987) striatal rCBF also have been observed. In thalamus, the majority reports no alterations (Catafau et al., 1994; Corson et al., 2002; Early et al., 1987; Parellada et al., 1994; Scottish Schizophrenia Research Group, 1998), although higher (Andreasen et al., 1997) and lower (Vita et al., 1995) perfusion also have been found. However, sex differences and a low number of participants may have influenced findings. First, healthy female subjects have higher global cerebral perfusion (Alisch et al., 2021; Gur et al., 1982; Liu et al., 2012; Mathew, Wilson, & Tant, 1986) and higher subcortical rCBF in thalamus and striatum compared with males (Ghisleni et al., 2015). However, it has not been investigated if rCBF in antipsychotic-naïve female patients with first-episode psychosis differ from rCBF in male patients. Second, the number of included participants has, in general, been small, and significant findings are seen in studies with few participants (*N* = 6–17). Thus, larger studies of antipsychotic-naïve patients are needed to clarify the role of striatal and thalamic rCBF alterations and possible sex differences in the psychosis pathophysiology.

Antipsychotic treatment is known to alter rCBF among others in striatum and thalamus. Studies of initially antipsychotic-naïve patients and HCs receiving various first- and second generation antipsychotic compounds consistently report increased striatal perfusion (Corson et al., 2002; Fernandez-Seara et al., 2011; Hawkins et al., 2018; Mehta et al., 2003; Michels, Scherpiet, Stampfli, Herwig, & Bruhl, 2016; Scottish Schizophrenia Research Group, 1998; Viviani, Graf, Wiegers, & Abler, 2013) as well as increases in striatal volume (Andersen et al., 2020). Cross-sectional studies of medicated patients also find increased perfusion in striatum and thalamus (Eisenberg et al., 2017; Kindler et al., 2018; Lahti, Weiler, Holcomb, Tamminga, & Cropsey, 2009; Miller et al., 1997a; Miller, Rezai, Alliger, & Andreasen, 1997b; Oliveira et al., 2018; Ota et al., 2014; Rodriguez et al., 1997; Xu et al., 2017; Zhu et al., 2015) as do studies of patients off-and-on antipsychotics (Eisenberg et al., 2017; Lahti et al., 2009). However, the effect of monotherapy with a partial dopamine agonist on rCBF has not been investigated.

The clinical correlation between rCBF and psychotic symptoms is also sparsely investigated. Some studies of previously medicated patients have found an association between rCBF changes in striatum and thalamus and improvement in positive symptoms (Eisenberg et al., 2017; Lahti et al., 2009), and striatal volume increases has also been related to treatment effect (Andersen et al., 2020). We recently reported that baseline measures of striatal dopaminergic activity and thalamic levels of glutamate were related to subsequent reduction of positive symptoms in a partly overlapping sample of initially antipsychotic-naïve patients (Bojesen et al., 2020; Sigvard et al., 2022). Brain perfusion is believed to reflect neuronal activity (Attwell et al., 2010), and baseline rCBF in striatum and thalamus may therefore also constitute markers of treatment outcome in antipsychotic-naïve patients.

To address these questions, we assessed rCBF before and after six weeks of monotherapy with a partial dopamine agonist in initially antipsychotic-naïve patients with first-episode psychosis and examined the associations with treatment outcome and sex. We tested the primary hypothesis that perfusion in striatum and thalamus would increase after treatment with a partial dopamine agonist and be higher in the antipsychotic-naïve state in female patients compared with male patients. Moreover, we explored if there was a different response to antipsychotic treatment in female patients compared with male patients. Our second hypothesis was that baseline striatal and thalamic perfusion would be related to reduction of positive symptoms after treatment.

## Participants and methods

### Participants

Antipsychotic-naïve patients with schizophrenia spectrum disorder and HCs were recruited from January 2014 to May 2019 as part of the larger, multimodal Pan European Collaboration on Antipsychotic Naïve Schizophrenia II (PECANSII) study previously described in detail (Bojesen et al., 2020). The study was approved by the Committee on Biomedical Research Ethics (H-3-2013-149), and all participants provided written informed consent after the study procedures were explained. Antipsychotic-naïve patients were recruited from mental health centers and outpatient services in the Capital Region of Denmark if the following inclusion criteria were fulfilled: a diagnosis of first-episode schizophrenia, schizoaffective disorder, or non-organic psychosis (ICD-10 criteria) as evaluated with the Schedules for Clinical Assessment in Neuropsychiatry (Wing et al., 1990), never treated with antipsychotic compounds or central nervous system stimulants (as reported by patients and confirmed by medical record), age between 18–45 years, and being legally competent. Patients were excluded if they had been treated with an antidepressant within the last 30 days or was involuntarily admitted. HCs recruited through online advertisement (www.forsøgsperson.dk) were matched on age, sex, and parental socioeconomic status, but were excluded if they fulfilled the criteria for clinical-high-risk of psychosis according to the Comprehensive Assessment of At-Risk Mental States (Yung et al., 1998), had a lifetime psychiatric diagnosis, or a first-degree relative with a psychiatric diagnosis. The following exclusion criteria applied for all participants: severe medical condition, previous head injury with unconsciousness >5 min, contraindication to magnetic resonance (MR) scans, and substance abuse in the past three months (current occasional substance use was tolerated for patients). Participants reported on their use of alcohol, nicotine, cannabis, other recreational drugs, and benzodiazepines and had a urine drug-screen test done (Rapid Response, Jepsen HealthCare, Tune, DK). In patients only, hemoglobin was assessed as part of routine blood tests at both visits. Occasional use of benzodiazepines was accepted for patients although not later than 12 h before examinations. After baseline examination, patients were treated with aripiprazole in individual dosing of 5–30 mg/day. Aripiprazole was a tool-compound chosen due to the common use in first-episode patients and the partial dopamine agonist properties. Other psychoactive compounds (antipsychotics, antidepressants, or mood stabilizers) were not permitted. If patients had to change antipsychotic compound prior to six weeks follow-up examinations due to inadequate clinical effect or severe side-effects, they were excluded.

Participants have been included in other publications described in the online Supplemental Methods, but rCBF data have not been reported previously.

### Clinical assessments

The Positive and Negative Syndrome Scale (PANSS) was used to assess psychopathology in patients by trained raters (Kay, Fiszbein, & Opler, 1987), and the minimum PANSS score was subtracted when calculating the percentage change in the PANSS positive score (Leucht, Davis, Engel, Kane, & Wagenpfeil, 2007). Level of function in all participants was assessed with the Personal and Social Performance Scale (PSP) (Morosini, Magliano, Brambilla, Ugolini, & Pioli, 2000).

### Magnetic resonance imaging acquisition

MR imaging was done as previously described (Bojesen et al., 2018) on a 3.0 Tesla Philips scanner with a 32-channel head coil. rCBF was acquired with the pseudo-Continuous Arterial Spin Labeling (PCASL) sequence (Dai, Garcia, de Bazelaire, & Alsop, 2008) with 30 pairs of perfusion weighted and control scans (dual echo EPI; 16 slices of 5 mm with an in-plane resolution of 3.55 × 3.55 mm^2^; SENSE factor 2.3; TR = 4100 ms; TE = 12 ms/28.5 ms at a post labeling delay of 1600 ms; labeling duration 1650 ms; background inversion pulses at 1663 and 2850 ms after the start of labeling). A reference scan was acquired to estimate the fully magnetized signal (M0): TR/TE = 10 s/9 ms. The PCASL sequence was obtained after approximately 45 min MR scanning preceded by a T1-weighted structural scan, spectroscopy sequences, and a functional resting state sequence. Motion issues were controlled at subject level as described in the online Supplementary Methods.

### Preprocessing of PCASL data

PCASL data were processed with the FSL software package (http://fsl.fmrib.ox.ac.uk/) using data acquired with the first echo. Extra-cerebral signal was removed from T1-weighted images with the ‘Brain extraction Tool’ (BET) and the raw PCASL scans were thereafter linearly co-registered with the skull-stripped T1-weighted image. The T1-weighted images were segmented with FAST from FSL software into gray- and white matter. Next, normalization of the T1-weighted image to Montreal Neurological Institute (MNI) standard space was performed. Maps of calibrated rCBF (ml/100 g/min) were calculated using tools provided by FSL (oxford_asl). Finally, rCBF maps were spatially transformed to MNI standard space, using a combination of the linear and non-linear transformation from anatomical data to MNI standard space.

#### Correction for gray and white matter partial volume effects

To account for possible inter-individual differences in gray and white matter, correction for gray and white matter partial volume effects was done using a linear regression model
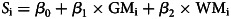
Where *S*_i_ is the scaled perfusion value of the *i*'th voxel within the region of interest (ROI) (such that 1 < *i* < *N*_r_, and *N*_r_ is the number of voxels in the ROI), GM_i_ is the partial volume fraction of gray matter, and WM_i_ the partial volume fraction of white matter at the same voxel. We used *β*_i_ as a measure of partial volume corrected regional gray matter perfusion. The value of *β*_0_ corresponds to perfusion in CSF and was held constant at 0.

#### Region of interest analyses of rCBF

A ROI approach was chosen to extract gray matter rCBF values from the primary ROIs striatum and thalamus and the following exploratory ROIs: striatal subdivisions (putamen, nucleus accumbens, and caudate), hippocampus, and frontal lobe. Global CBF was estimated by averaging across the whole brain mask. ROIs from subcortical structures were defined by segmenting the structural T1 scan using FreeSurfer v. 6.0.0 (https://surfer.nmr.mgh.harvard.edu/) by applying individual anatomical regions to perfusion maps in structural space (subject space). The cortical MNI 152 atlas from FSL was used to estimate rCBF in frontal lobe.

#### Voxel-wise analysis of rCBF

Explorative whole brain voxel-wise analyses were performed to support the ROI analyses and explore rCBF differences at baseline and after treatment with age, sex, and global perfusion as co-variates and with correction for partial volume effects. Voxel-wise analyses were performed with and without masking deep white matter. The ‘randomise’ program from the FMRIB Software Library version (www.fmrib.ox.ac.uk/) using non-parametric, permutation-based statistical inference and threshold-free cluster enhancement was used. The threshold for statistical maps was set *p* < 0.05 (corrected for multiple comparisons).

### Statistics

Demographic variables and clinical characteristics were compared with χ^2^, Fisher's exact test, or dependent and independent *t* tests as appropriate.

The following co-variates of no interest were included in all analyses: global rCBF (to eliminate global effects on regional rCBF), age and sex as well as smoking status that impact rCBF (Alisch et al., 2021; Elbejjani et al., 2019). Hemoglobin was adjusted for in post-hoc analyses of sex-differences in the patient group but was not available in HCs.

Baseline differences in rCBF were investigated with a general linear model (GLM) including a sex × group interaction. In cases with a significant interaction, post hoc analyses evaluated differences between female and male patients (main effect of sex). Differences between female and male HCs are reported as exploratory outcomes in the online Supplementary Results.

The primary hypothesis that treatment with a partial dopamine agonist would increase perfusion in striatum and thalamus was tested in a linear mixed model, where a group × time interaction evaluated the treatment effect. In cases with a significant group × time interaction, post hoc tests evaluated group differences after six weeks. Sex differences in the rCBF trajectory were tested by replacing the interaction term with a group × time × sex interaction. In cases with significant interactions, post hoc analyses of the patient group only evaluated a different trajectory between male and female patients by using the interaction term sex × time for the patient group in a GLM.

The statistical models are described in detail in the online Supplementary Methods.

The associations between reduction in PANSS positive after treatment and baseline rCBF or rCBF after treatment were evaluated in GLMs with the rCBF change being calculated as: (rCBF after treatment – rCBF before treatment)/ rCBF before treatment.

The significance level was adjusted to *p* = 0.025 due to two primary ROIs.

Statistical analyses were performed using SAS version 7.1 (SAS institute, Cary, NC).

## Results

At baseline, 52 patients (58% females) with schizophrenia (*N* = 39), schizoaffective disorder (*N* = 1), or non-organic psychosis (*N* = 12) and 57 HCs (63% females) were included and after six weeks, 32 patients and 53 HCs were re-examined. Three patients and seven HCs did not have a usable pCASL scan at baseline and were excluded from the analyses, and 20 patients and one HCs were excluded after six weeks as specified in online Supplementary Fig. S1. Demographic and clinical characteristics of the remaining 49 patients and 50 HCs at baseline and 29 patients and 49 HCs after six weeks are provided in Table 1. There were no differences between groups with regards to age, parental socioeconomic status, and current cannabis use, but patients had significantly fewer years of education, smoked more, and had lower level of function than HCs as expected (Table 1). PANSS positive score significantly decreased after treatment (Table 1).

**Table 1.** Demographics and clinical characteristics

	Baseline			Follow-up		
	FEP	HCs	Statistics	FEP	HCs	Statistics
Number of participants^a^: *N* (F/M)	49 (29/31)	50 (31/19)	χ^2^ = 0.08, *p* = 0.77	29 (16/13)	49 (31/18)	χ^2^ = 0.50, *p* = 0.48
Age ± s.d., years	22.6 ± 5.2	22.3 ± 4.4	*T*(105) = 0.37, *p* = 0.71			
Parental socioeconomic status (high/moderate/low/missing)	13/29/6/1	17/29/4/0	Fisher's *p* = 0.66			
Education ± s.d., years	12.1 ± 2.2	14.1 ± 2.5	*T*(97) = −4.16, *p* < 0.001			
Ethnicity (*n*) White/Asian/Middle east	46/2/1	50/0/0				
Duration of untreated psychosis, Median (25–75th percentile) in weeks	26.0 (12.5–117.0)					
Current tobacco use, yes/no/missing	18/31	4/46	χ^2^ = 11.82, *p* < 0.001	11/18/0	7/41/1	χ^2^ = 5.50, *p* = 0.019
Current cannabis use, yes/no/missing	3/46/0	2/43/0	Fisher's *p* = 0.68	0/29/0	0/42/7	NA
PANSS Positive, mean ± s.d. (% decrease)^b^	18.6 ± 4.4	–		13.3 ± 4.5 (42.2 ± 31.9%)	–	*T*(28) = 7.81, *p* < 0.001^c^
PANSS Negative, mean ± s.d. (% decrease)^b^	19.6 ± 5.5	–		17.3 ± 5.7 (14.1 ± 72.8%)	–	*T*(28) = 3.21, *p* = 0.003^c^
PANSS General, mean ± s.d. (% decrease)^b^	36.6 ± 7.4	–		29.2 ± 7.0 (31.4 ± 35.1%)	–	*T*(28) = 6.19, *p* = 0.001^c^
PANSS Total, mean ± s.d. (% decrease)^b^	75.0 ± 14.5	–		60.3 ± 14.8 (28.2 ± 51.4%)	–	*T*(28) = 7.01, *p* < 0.001^c^
Clinical global impression, ± s.d., (Improvement ± s.d.)	4.3 ± 0.6 (NA)	–		3.7 ± 0.5 (2.3 ± 0.6)	–	*T*(25) = 5.72, *p* < 0.001^c^
PSP score, mean ± s.d.	48.0 ± 12.6	89.5 ± 3.5	*T*(93) = −21.48, *p* < 0.001	58.1 ± 12.5	90.1 ± 3.5	*T*(72) = −20.95, *p* < 0.001
Benzodiazepines (daily/per needed/no)	1/4/44	0/0/50	Fisher's *p* = 0.03	2/1/26	0/0/49	Fisher's *p* = 0.05

Abbreviations: FEP, First-episode psychosis patients; HCs, Healthy controls; F, Females; M, Males; s.d., Standard deviation; PANSS, Positive and negative syndrome scale; PSP, Personal and Social Performance scale.

a *N* states the number of participants with a usable pseudo-Continuous Arterial Spin Labeling scan.

b Minimum PANSS score was subtracted before calculating the percentage change.

c Statistics represent patient values at baseline compared with follow-up after six weeks.

The median dose of aripiprazole (25th–75th percentile) after six weeks was 10 mg (5.0–15.0 mg), and median serum aripiprazole level was 169.9 *μ*g/l (111.2–246.6 *μ*g/l) suggesting that patients were treated with sufficient doses and were compliant (Sparshatt, Taylor, Patel, & Kapur, 2010).

### Resting CBF in antipsychotic-naïve patients at baseline

#### Region of interest analyses

*Striatum*: Striatal perfusion did not differ between patients and HCs at baseline (*p* = 0.99). There was a significant sex × group interaction (*p* = 0.014) and post hoc tests revealed that female patients had significantly lower striatal rCBF than male patients (*p* = 0.009).

*Thalamus*: Thalamic perfusion did not differ between patients and HCs at baseline (*p* = 0.79) and the sex × group interaction did not reach significance (*p* = 0.06).

*Explorative regions:* In the explorative regions of interest there were no significant group differences at baseline in nucleus accumbens (*p* = 0.41), caudate (*p* = 0.64), putamen (*p* = 0.55), hippocampus (*p* = 0.24), frontal lobe (*p* = 0.80), or in global rCBF (*p* = 0.58). The sex × group interaction was borderline significant for nucleus accumbens (*p* = 0.027), but the main effect of sex was not significant for the patients (*p* = 0.08). For the remaining regions, the sex × group interaction was insignificant (*p* = 0.11–0.82).

#### Voxel-wise analyses

Explorative voxel-wise analyses revealed no significant group differences at baseline.

### rCBF after six weeks of treatment with partial dopamine agonism

#### Region of interest analyses

**Fig. 1.**
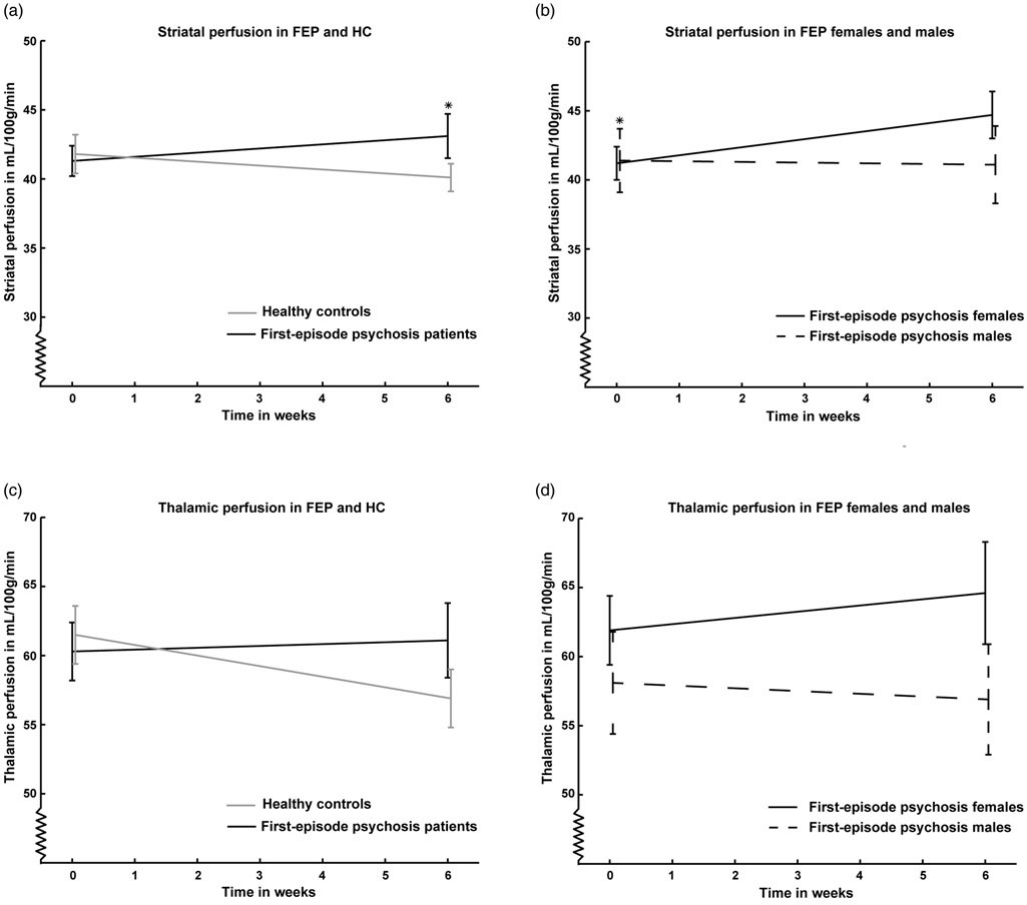
Figure 1 shows mean resting cerebral blood flow (rCBF) in mL/100 g/min in striatum (a) and thalamus (c) in first-episode patients (black line) before and after six weeks of monotherapy with a partial dopamine agonist compared with healthy controls (gray line) as well as rCBF in female patients (black line) compared with male patients (black dashed line) in striatum (b) and thalamus (d). A: Striatal rCBF was affected by treatment in patients (group × time: *p* = 0.020) due to significantly higher rCBF in first-episode patients after treatment compared with healthy controls. B: Striatal rCBF was significantly lower in female patients compared with male patients at baseline. C: Thalamic rCBF was affected at trendlevel (group × time: *p* = 0.040) but did not differ significantly between patients and healthy controls at baseline or after treatment. D: Thalamic rCBF did not differ significantly between female and male patients. Vertical bars represent standard error of the mean. *: *p* < 0.025 (adjusted for two regions). Abbreviations: FEP, first-episode patients with psychosis; HC, Healthy controls.

*Striatum*: The rCBF change from baseline to six weeks was significantly different in patients compared with HCs (significant group × time) due to increased rCBF in patients after treatment as summarized in Table 2 and Fig. 1a. There was a different trajectory in females compared to males (significant group × time × sex interaction: *p* = 0.020) and post hoc tests of the patient group only revealed a borderline significant different trajectory in female and male patients (month × sex: *p* = 0.025) due to lower rCBF at baseline in the female patients (*p* = 0.009) but no sex-difference after six weeks (*p* = 0.75; Fig. 1b).

**Table 2.** Resting cerebral blood flow before and after treatment with a partial dopamine agonist in primary and explorative regions of interest

Regions of interest	Mean CBF (mL/100 g/min ± s.d.)						Group × time interaction	Post hoc tests	Main effect group
	*N* Baseline/*N* 6weeks		Baseline		6 weeks				
	FEP	HC	FEP	HC	FEP	HC			
Thalamus (%change ± s.d.)	49/29	50/49	60.3 ± 14.6	61.5 ± 15.0	61.1 ± 14.8 (3.0 ± 20.9%)	56.9 ± 14.7 (−6.4 ±2 0.7%)	*p* = 0.040^a^	FEP = HC baseline: *p* = 0.89 FEP > HC 6 weeks: *p* = 0.14	–
Whole striatum^b^ (%change ± s.d.)	46/29	48/47	41.3 ± 7.8	41.8 ± 9.4	43.1 ± 8.5 (4.8 ± 18.4%)	40.1 ± 6.9 (−0.7±24.0%)	*p* = 0.020^a^	FEP = HC baseline: *p* = 0.92 FEP > HC 6 weeks: *p* = 0.006	–
Putamen^b^ (%change ± s.d.)	46/29	48/47	42.4 ± 8.8	43.8 ± 10.9	45.0 ± 8.9 (7.8 ± 22.4%)	41.6 ± 7.7 (−2.5 ± 22.6%)	*p* = 0.004	FEP = HC baseline: *p* = 0.64 FEP > HC 6 weeks: *p* = 0.005	–
Nucleus accumbens^b^ (%change ± s.d.)	46/29	48/47	36.2 ± 9.0	37.5 ± 12.3	40.0 ± 10.2 (16.0 ± 32.9%)	35.7 ± 8.8 (2.1 ± 28.2%)	*p* = 0.01	FEP = HC baseline: *p* = 0.51 FEP > HC 6 weeks: *p* = 0.04	–
Caudate^b^ (%change ± s.d.)	46/29	48/47	43.9 ± 9.6	43.6 ± 9.9	45.4 ± 10.5 (4.5 ± 21.1%)	42.8 ± 8.3 (2.2 ± 29.6%)	*p* = 0.46	–	*p* = 0.26
Hippocampus (%change ± s.d.)	49/29	50/49	30.7 ± 7.5	32.1 ± 9.8	31.6 ± 7.5 (7.8 ± 22.4%)	29.8 ± 6.5 (−2.5 ± 22.6%)	*p* = 0.13	–	*p* = 0.99
Frontal lobe (%change ± s.d.)	49/29	50/49	56.7 ± 57.2	57.2 ± 11.4	55.7 ± 9.9 (−1.3 ± 14.4%)	54.8 ± 10.4 (−2.6 ± 18.3%)	*p* = 0.70	–	*p* = 0.82
Global rCBF^c^ (%change ± s.d.)	49/29	50/49	50.7 ± 8.4	51.4 ± 9.3	49.6 ± 8.1 (−2.1 ± 13.2%)	48.7 ± 8.3 (−3.7 ± 17.4%)	*p* = 0.42	–	*p* = 0.95

Abbreviations: FEP, First-episode psychosis patients; HC, Healthy controls; rCBF, resting cerebral blood flow; s.d., standard deviation.

Table 2 shows resting cerebral blood flow (rCBF) in regions of interest in first-episode patients and healthy controls. Statistical analyses are adjusted for global rCBF, smoking status, age, and sex.

a The significance level was set to *p* < 0.025 due to two primary regions of interest.

b Striatal rCBF could not be calculated in three patients due to poor anatomical segmentation of striatum.

c Analyses of global rCBF are corrected for age and sex. The percent change of rCBF was calculated as: [(baseline-6 weeks)/baseline] × 100%.

*Thalamus*: There was a trend for a difference in rCBF change from baseline to six weeks in patients compared with HCs (group × time: *p* = 0.04, Table 2 and Fig. 1c). There was a significant different trajectory in females compared to males (group × time × sex interaction: *p* = 0.024) and post hoc test of the patient group only revealed a trend for a different trajectory in female and male patients (month × sex: *p* = 0.05) but not significant differences at baseline (*p* = 0.11) or after six weeks (*p* = 0.91).

*Explorative regions*: The rCBF change from baseline to six weeks was significantly different in patients compared with HCs for nucleus accumbens and putamen due to increased rCBF in patients after treatment, whereas the other explorative ROIs were unaffected by treatment (Table 2). For nucleus accumbens there was a significant group × time × sex interaction (*p* = 0.011) and post hoc tests of the patients group revealed a borderline significant month × sex interaction (*p* = 0.026) due to a trend for lower rCBF in female patients at baseline. For putamen, the group × time × sex interaction was borderline significant (*p* = 0.029), but post hoc tests were insignificant (month × sex: *p* = 0.27). The group × time × sex interaction was insignificant for the caudate (*p* = 0.56), hippocampus (*p* = 0.60), frontal lobe (*p* = 0.22), and global rCBF (*p* = 0.56).

The effect of treatment on rCBF without adjustment for global perfusion is provided in online Supplementary Table S1 and analyses without adjustment for any co-variates in online Supplementary Table S2.

#### Voxel-wise analyses

**Fig. 2.**
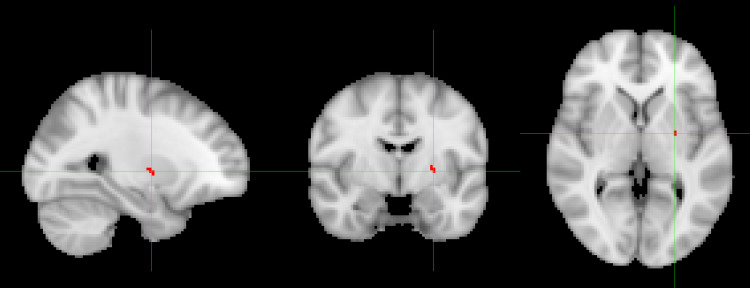
Figure 2 shows increased perfusion in putamen in initially antipsychotic-naïve patients with psychosis after six weeks monotherapy with aripiprazole as compared with healthy controls (*p* < 0.05 based on permutation-based analysis corrected for multiple comparisons) in a voxel-wise analysis, where white matter was masked. The red color illustrates the significance level *p* < 0.05.

Explorative voxel-wise analyses without masking deep white matter revealed significantly increased rCBF in patients after six weeks of treatment in putamen, caudate, and white matter in frontal lobe when compared with HCs as shown in online Supplementary Fig. S3. When masking deep white matter, the CBF maps also revealed a striatal increase in perfusion after treatment although in a more restricted area as illustrated in Fig. 2.

### Baseline rCBF and symptom improvement after treatment

Striatal and thalamic perfusion at baseline was not associated with improvement in PANSS positive symptoms (striatum: *p* = 0.32; thalamus: *p* = 0.33). For the exploratory ROIs, there was a significant negative association between nucleus accumbens baseline perfusion and improvement in PANSS positive (*p* = 0.046), whereas the associations for the other ROIs were insignificant (*p* = 0.14–0.68).

Analyses without the co-variates age, sex, and global rCBF revealed significant negative associations between improvement in PANSS positive symptoms and perfusion in whole striatum (*p* = 0.015), nucleus accumbens (*p* = 0.006), putamen (*p* = 0.02), and caudate (*p* = 0.005) but not for any other ROIs (*p* = 0.09–0.74).

There was no evidence for sex-specific associations between CBF and improvement in PANSS positive (CBF × sex interactions for all ROIs insignificant: *p* = 0.26–0.81).

Exploratory analyses revealed no association between baseline rCBF in any of the ROIs and improvement in PANSS total (*p* = 0.36–0.93), PANSS general (*p* = 0.46–0.98), or PANSS negative (*p* = 0.21–0.94).

### rCBF changes after treatment and symptom improvement

Changes in striatal and thalamic perfusion after treatment were not associated with improvement in PANSS positive score (striatum: *p* = 0.97; thalamus: *p* = 0.72; without co-variates: striatum: *p* = 0.94; thalamus: *p* = 0.92). Also, there was no significant associations between changes in rCBF in explorative ROIs and improvement in PANSS positive score (*p* = 0.18–0.96; without co-variates: *p* = 0.35–0.94).

The CBF × sex interactions for all ROIs were insignificant (*p* = 0.07–0.43).

Exploratory analyses revealed no association between changes in rCBF in any of the ROIs and improvement in PANSS total (*p* = 0.41–0.98), PANSS general (*p* = 0.21–0.87), or PANSS negative (*p* = 0.60–0.95). There was a trend for a negative association between aripiprazole serum levels and rCBF change in nucleus accumbens (*p* = 0.06) but not in any other ROI (*p* = 0.26–0.99).

## Discussion

This study is the first to investigate striatal and thalamic perfusion in a large group of initially antipsychotic-naïve psychosis patients before and after monotherapy with a partial dopamine receptor agonist. The main finding is that partial dopamine agonism increases striatal perfusion. Moreover, findings suggest that treatment affects thalamic perfusion to a lesser degree, and that female patients have lower striatal perfusion than males from illness onset. Last, lower baseline rCBF in nucleus accumbens was related to a greater improvement of positive symptoms after treatment.

Our finding of no striatal rCBF alterations in the entire group of antipsychotic-naïve patients is in line with existing studies (Andreasen et al., 1997; Catafau et al., 1994; Corson et al., 2002; Early et al., 1987; Parellada et al., 1994; Scottish Schizophrenia Research Group, 1998; Vita et al., 1995) but at odds with the theory of striatal overactivity as a driver of psychosis (Glenthoj & Hemmingsen, 1997; Howes & Murray, 2014; Kapur, 2003). This discrepancy might be explained by sex differences since we observed lower striatal perfusion in antipsychotic-naïve female patients. However, the lower rCBF in female patients contrasts with our hypothesis based on healthy females with higher striatal rCBF compared with males (Ghisleni et al., 2015). It is therefore likely that reduced rCBF is part of the psychosis pathophysiology in female patients. Moreover, it is possible that striatal hyperactivity primarily is part of psychosis pathophysiology in male patients. In support, estrogen is believed to increase striatal dopaminergic sensitivity in females (Yoest, Quigley, & Becker, 2018) and this may cause a sensitized striatal dopaminergic system prone for development of psychotic symptoms without measurable striatal overactivity at illness onset in females. In line with this, we previously found a significant association between psychotic symptoms and measures of presynaptic dopamine activity in an overlapping sample of antipsychotic-naïve psychosis patients with a majority of females (66%) without detecting differences in dopamine synthesis between patients and HCs (Sigvard et al., 2022). These potential sex differences are clinically relevant to investigate further. For example, females require lower serum levels of antipsychotics to reach striatal dopamine receptor occupancy (Brand et al., 2021; Hoekstra et al., 2021) and female-specific description guidelines may therefore be needed.

Thalamic perfusion was not increased at baseline in the antipsychotic-naïve patients corresponding to findings in previous rCBF studies (Catafau et al., 1994; Corson et al., 2002; Early et al., 1987; Parellada et al., 1994; Scottish Schizophrenia Research Group, 1998). However, it contrasts our previously findings of increased thalamic glutamate levels in an overlapping cohort of antipsychotic-naïve patients with psychotic disorder (Bojesen et al., 2020, 2021). Glutamatergic neurotransmission is believed to increase perfusion (Attwell et al., 2010) as confirmed in a previous *in vivo* study that reported a positive associations between glutamate levels and rCBF in HCs (Bojesen et al., 2018). Based on these findings, one would expect thalamic perfusion in antipsychotic-naïve patients to be increased from illness onset as well. However, perfusion is regulated by many neurotransmitters and is among others reduced by activity of GABAergic interneurons (Franklin et al., 2011; Krause et al., 2014) that also are found in thalamus (de Biasi, Frassoni, & Spreafico, 1986). It is possible that the net effect of increased glutamatergic activity and a possible compensatory increased GABAergic activity in thalamus may be no alterations in thalamic perfusion.

Treatment with a partial dopamine receptor agonist for six weeks significantly increased striatal rCBF and affected thalamic perfusion as also seen in patients treated with first- and second generation antipsychotics (Eisenberg et al., 2017; Kindler et al., 2018; Lahti et al., 2009; Miller et al., 1997b; Scheef et al., 2010; Xu et al., 2017; Zhu et al., 2015). This suggests that not only dopamine D2 antagonism but also partial dopamine D2 agonism increases metabolism after short term treatment in striatum and thalamus that are regions believed implicated in psychotic disorder (Carlsson, Waters, & Carlsson, 1999). The effect of treatment with a partial dopamine agonist on striatal perfusion was most robust, as it was found in both ROI and voxel-wise analyses. Global perfusion impacts regional perfusion in between-group studies (Selvaggi et al., 2022; Turkheimer et al., 2020; Viviani et al., 2009), but, despite this, we found significantly increased striatal perfusion both with and without adjustment for global perfusion supporting the robustness of this finding. However, the magnitude of the striatal rCBF increase after partial dopamine agonism might have been less pronounced than after dopamine antagonism, as preclinical studies have found that dopamine D2/D3 receptor antagonism increases while agonism decreases striatal cerebral blood volume (Sander et al., 2013; Sander, Hooker, Catana, Rosen, & Mandeville, 2016).

Thalamic perfusion after treatment was only affected in the ROI analyses and only at trend level. This suggests that the effect of partial dopamine receptor agonism is most pronounced in striatum, which might be explained by a higher concentration of D2 receptors in this region (Selvaggi et al., 2019). However, studies of the long-term effect of treatment on thalamic rCBF are needed, as thalamic rCBF is reduced in medicated patients (Walther et al., 2011).

We did not find an association between baseline rCBF in whole striatum and reduction of positive symptoms after treatment but found a significant negative association in nucleus accumbens. This is in line with our previous finding of an association between baseline striatal decarboxylation rate and improvement in positive symptoms in an overlapping cohort of patients (Sigvard et al., 2022). Interestingly, the change in perfusion was not related to treatment effect, suggesting baseline perfusion as a better predictor for subsequent outcome.

We had expected an association between baseline perfusion in thalamus and symptoms improvement given our previous finding of an association between baseline thalamic glutamate levels and treatment outcome in an overlapping cohort (Bojesen et al., 2020). This may suggest that more direct measures of neurochemical function are better predictors than rCBF of treatment effect.

Last, it should be noted that rCBF might be related to other symptom dimensions in psychosis, as associations with formal thought disorders, altered affectivity, and abnormal motor behavior, among others, previously have been reported (Horn et al., 2009; Kindler et al., 2019; Stegmayer et al., 2017; Walther et al., 2017).

Frontal rCBF both with and without adjustment for global rCBF was not reduced in the antipsychotic-naïve state as found in a previous study of first-episode patients free from antipsychotic medication (Selvaggi et al., 2022), and treatment did not affect rCBF in frontal lobe as suggested by previous studies (Goozee et al., 2014). This might indicate that frontal changes in perfusion only occur in subgroups from illness onset and is affected by treatment later in the illness. It is also possible that previous studies have assessed a combination of gray- and white matter perfusion since we found increased white matter rCBF in frontal lobe in the voxel-wise analysis when not masking white matter. Specialized ASL sequences can provide reliable white matter rCBF measures (Giezendanner et al., 2016), but the sensitivity is considered too small in sequences optimized for gray matter rCBF (Alsop et al., 2015).

Last, perfusion in the explorative ROI hippocampus was also not increased from illness onset as found in studies of patients at UHR for psychosis and subjects scoring high on schizotypy (Allen et al., 2016, 2018; Modinos et al., 2018), which might be due to diagnostic heterogeneity between these populations and patients with first-episode psychosis.

Strength of the current study is a large group of initially antipsychotic-naïve patients treated with a partial dopamine D2 receptor agonist as monotherapy, but limitations should also be addressed. Most importantly, post hoc analyses of sex differences in patients after treatment might have lacked power due to drop-out after baseline (*N* = 29).

In conclusion, increased striatal rCBF is induced by antipsychotic treatment with a partial dopamine agonist as also seen after dopamine antagonism, and female patients have lower striatal perfusion from illness onset. Moreover, rCBF in nucleus accumbens at illness onset is related to treatment effect. The findings stress the importance of sex differences and treatment effects when investigating the neurobiology of psychosis.

## Supplementary material

For supplementary material accompanying this paper visit https://doi.org/10.1017/S0033291723000144. click here to view supplementary material
