# Supplementary material for: Cerebral blood flow in striatum is increased by partial dopamine agonism in initially antipsychotic-naïve patients with psychosis
Source: Psychol Med. 2023 Feb 9;53(14):6691–701. doi: 10.1017/S0033291723000144 (PMC10600821; doi:10.1017/S0033291723000144)
Supplement: Supplementary file 1 [file S0033291723000144sup001.docx]

**Supplementary information**

**Supplementary Participants and Methods**

**Supplementary** **Figure 1: Flowchart of the study**
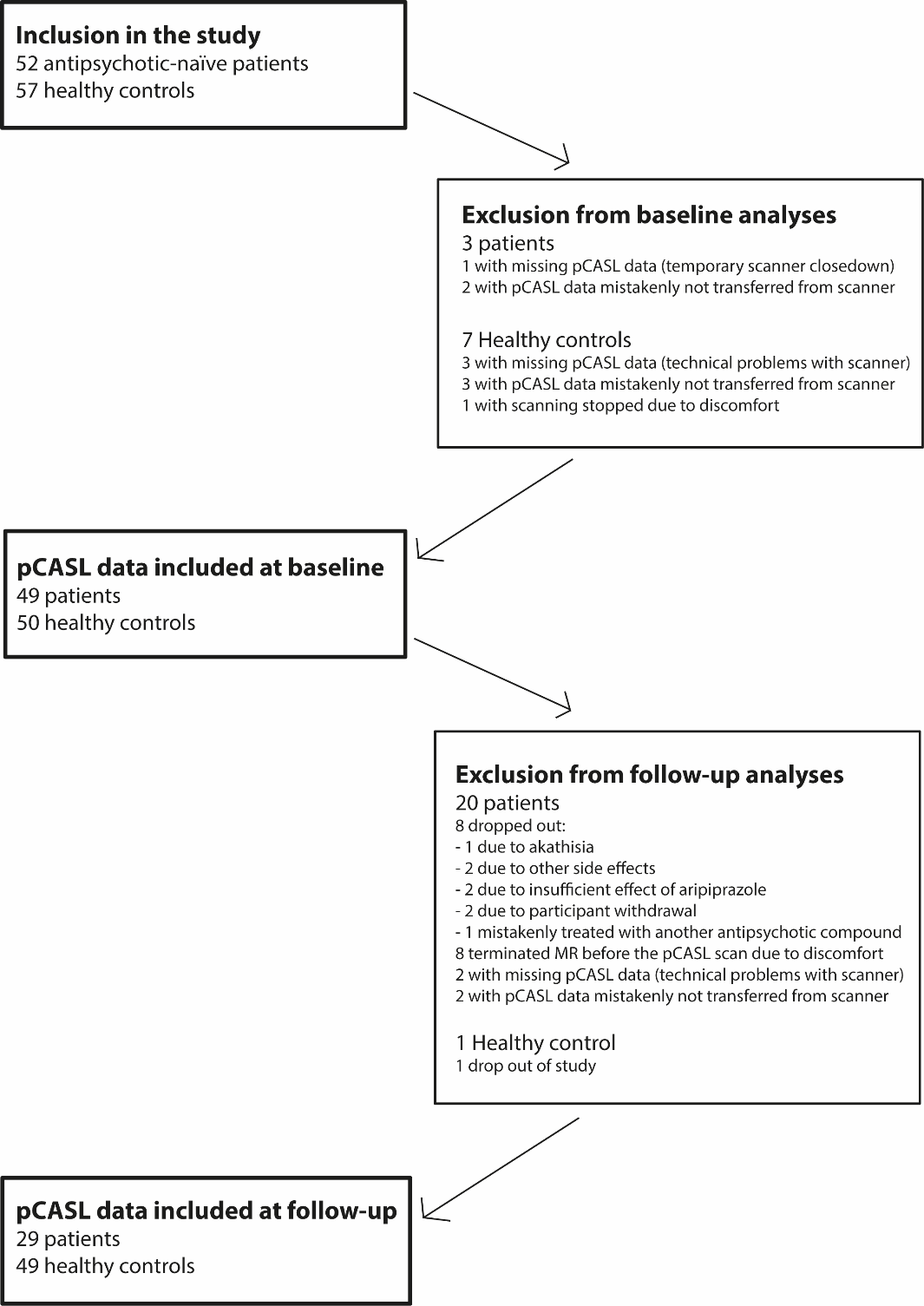


Supplementary Figure 1 shows the number of included participants and exclusion from baseline and follow-up analyses. pCASL: pseudo-Continuous Arterial Spin Labelling.

**Participants and Methods**

**Participants**

Participants have been included in other publications (Bojesen *et al.*, 2021, Bojesen *et al.*, 2020, Fagerlund *et al.*, 2021, Jessen *et al.*, 2019, Nielsen *et al.*, 2022, Sigvard *et al.*, 2022, Tangmose K, 2020), but rCBF data have not been reported previously.

**Magnetic resonance imaging acquisition**

Motion issues were controlled at subject level using the outlier detection method provided by the oxford_asl software. Following this algorithm outlier frames are detected using the "dvars" parameter (i.e. root mean square (RMS) intensity difference between sequential frames) and removed if exceeding a certain threshold. In general, 0 to 6 outlier frames were removed from each dataset of 30 frames with the median value was 1 frame removed as illustrated in Supplementary Figure 2. We chose not to calculate and correct for motion parameters as such, since these may be affected by the alternating contrast of the tag-control pairs (see also <https://fsl.fmrib.ox.ac.uk/fsl/fslwiki/FSLMotionOutliers>).

**Supplementary Figure 2**

**A**


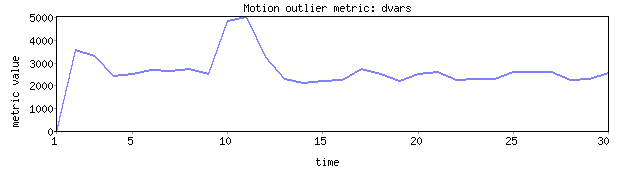


**B**


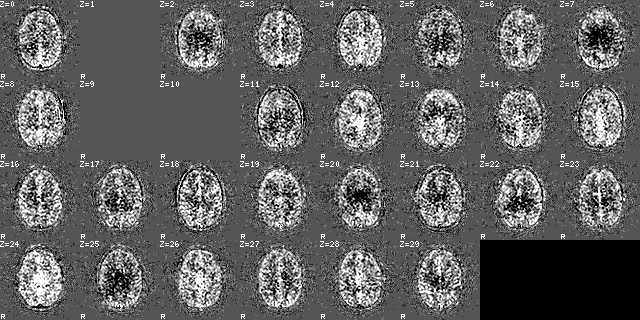


Supplementary Figure 2 illustrates the outlier detection method provided by the oxford_asl software. Outlier frames were detected with the ‘dvars’ parameter using the root mean square (RMS) intensity difference between sequential frames as illustrated in A. Frames were removed if they exceeded a certain threshold as illustrated in B. Note that 1 on the x-axis in A corresponds to Z=0 in B. Abbreviation: Dvars (D referring to temporal derivative of timecourses, VARS referring to RMS variance over voxels). RMS: Root mean square.

**Statistics**

Baseline differences in rCBF were investigated with a general linear model (GLM) for patients compared with healthy controls in the following model:

rCBF = β_0_*group + β_1_* age + β_2_* sex + β_3_* smoking status + β_4_* global rCBF + β_5_* group*sex

In cases with a significant sex*group interaction post hoc analyses were performed with group differences between female and male patients as primary outcome in the following model:

rCBF in the patient group = β_0_* sex + β_1_* age + β_2_* smoking status + β_3_* global rCBF

where the main effect of sex evaluated if rCBF was different in antipsychotic-naïve first-episode female patients compared with male patients. In exploratory post-hoc analyses, a similar model evaluated sex-differences in healthy control females compared with males at baseline.

The primary hypothesis that treatment with a partial dopamine agonist would increase perfusion in striatum and thalamus was tested in the following linear mixed model, where a significant group*time interaction evaluated the treatment effect:

rCBF = β_0_*group + β_1_*time + β_2_*age + β_3_*sex + β_4_*global rCBF + β_5_*smoking status + β_6_*group*time

In cases with a significant group*time interaction, explorative post hoc tests evaluated group differences after six weeks and in the absence of significant interactions, the model was re-evaluated without the interaction terms.

Sex differences in the rCBF trajectory was tested in a similar model by replacing the interaction term above with a group*time*sex interaction in the model below:

rCBF = β_0_*group + β_1_*time + β_2_*age + β_3_*sex + β_4_*global rCBF + β_5_*smoking status + β_6_*group*time*sex

In cases with significant interactions, post hoc analyses of the patient group only evaluated if there was a different trajectory of rCBF after treatment in female patients compared with male patients by using the following model:

rCBF (patients only) = β_0_*time + β_1_*age + β_2_*sex + β_3_*smoking status + β_4_*global rCBF + β_5_*time*sex

**Supplementary Results**

**Sex differences in rCBF in healthy controls at baseline**

*Striatum*: There was a significant sex*group interaction (p=0.014), but post hoc tests revealed no sex-difference in the HCs at baseline (p=0.41).

*Thalamus*: The sex*group interaction did not reach significance (p=0.06).

*Explorative regions*: The sex*group interaction was significant for nucleus accumbens (p=0.027), but in the post hoc analyses there were no main effect of sex in the HCs (p=0.22). For the remaining regions, the sex*group interactions were insignificant (p=0.11-0.82).

*Voxel-wise analyses without masking deep white matter*

Explorative voxel-wise analyses without masking deep white matter revealed significantly increased rCBF in patients after six weeks of treatment in putamen, caudate, and white matter in frontal lobe when compared with HCs as shown in supplementary Figure 3.

**Supplementary Figure 3**


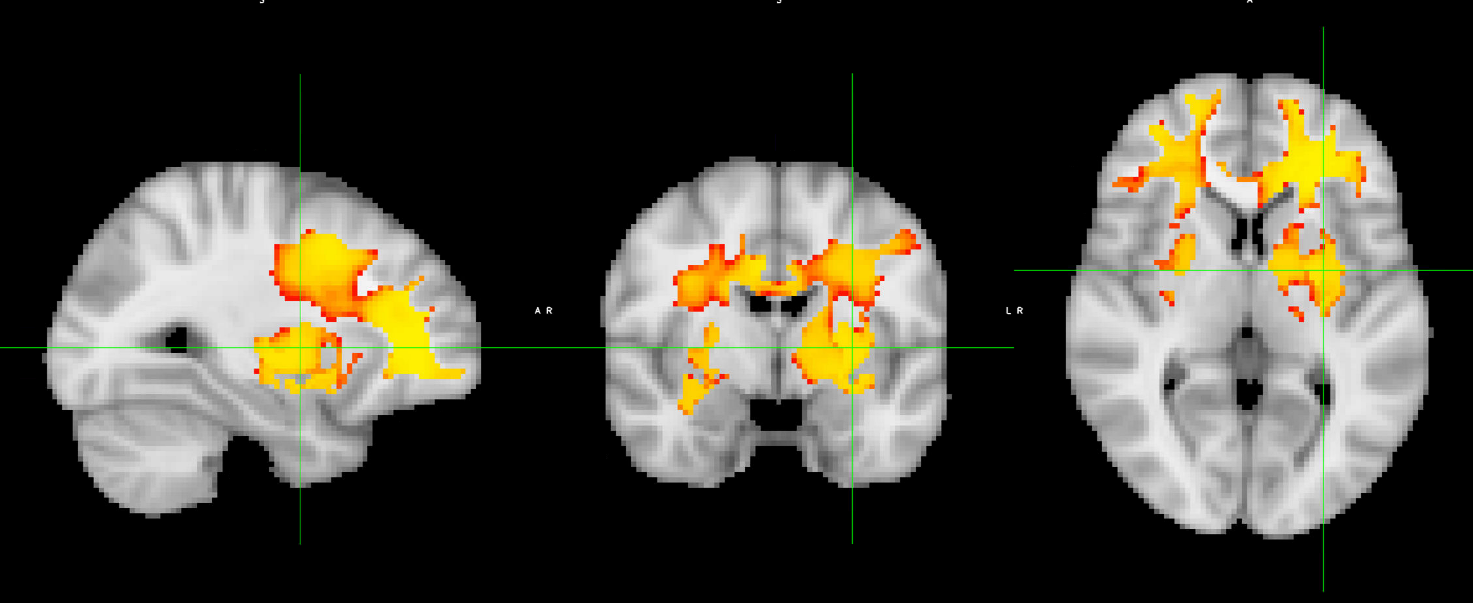


Supplementary Figure 3 shows increased perfusion in putamen, caudate, and frontal white matter in initially antipsychotic-naïve patients with psychosis after six weeks monotherapy with aripiprazole as compared with healthy controls (p<0.05 based on permutation-based analysis corrected for multiple comparisons). Colors illustrate the significance ranging from red (p<0.05) to yellow (p<0.005).

**Supplementary Table S1: Resting cerebral blood flow before and after treatment with adjustment for age, sex, and smoking status**

| **Regions of interest** | **Baseline** | | **6 weeks** | | **Interaction** | **Post hoc tests** | **Main effect of group** |
| --- | --- | --- | --- | --- | --- | --- | --- |
|  | **FEP** | **HC** | **FEP** | **HC** |  |  |  |
| **Thalamus rCBF**  in mL/100g/min ± SD | 60.3±14.6 | 61.5±15.0 | 61.1±14.8 | 56.9±14.7 | p=0.022 | FEP = HC baseline: p=0.91  FEP > HC 6 weeks: p=0.05 | - |
| **Whole striatum**  in mL/100g/min ± SD | 41.3±7.8 | 41.8±9.4 | 43.1±8.5 | 40.1±6.9 | P=0.013 | FEP = HC baseline: p=0.85  FEP > HC 6 weeks: p=0.04 |  |
| **Putamen rCBF**  in mL/100g/min ± SD | 42.4±8.8 | 43.8±10.9 | 45.0±8.9 | 41.6±7.7 | p=0.022 | FEP = HC baseline: p=0.64  FEP > HC 6 weeks: p=0.04 | - |
| **Nucleus accumbens rCBF**  in mL/100g/min ± SD | 35.6±9.7 | 37.5±12.3 | 40.0±10.2 | 35.7±8.8 | p=0.009 | FEP = HC baseline: p=0.54  FEP > HC 6 weeks: p=0.06 | - |
| **Caudate rCBF**  in mL/100g/min ± SD | 43.9±9.6 | 43.6±9.9 | 45.4±10.5 | 42.8±8.3 | p=0.34 | - | p=0.55 |
| **Hippocampus rCBF**  in mL/100g/min ± SD | 30.7±7.5 | 32.1±9.8 | 31.6±7.5 | 29.8±6.5 | p=0.08 | - | p=0.74 |
| **Frontal lobe rCBF**  in mL/100g/min ± SD | 56.7±57.2 | 57.2±11.4 | 55.7±9.9 | 54.8±10.4 | p=0.52 | - | p=0.96 |
| **Global rCBF**  in mL/100g/min ± SD | 50.7±8.4 | 51.4±9.3 | 49.6±8.1 | 48.7±8.3 | p=0.42 | - | p=0.95 |

Supplementary Table S1 shows regional cerebral blood flow in regions of interest in initially antipsychotic-naïve patients with schizophrenia or psychosis and matched healthy controls in analyses adjusted for age, sex, and smoking status, but not global rCBF.

Abbreviations: FEP: First-episode patients with psychosis or schizophrenia; HC: Healthy controls; rCBF: resting cerebral blood flow; SD; standard deviation.

**Supplementary Table S2: Resting cerebral blood flow before and after treatment in primary and explorative regions of interest**

| **Regions of interest** | **Baseline** | | **6 weeks** | | **Interaction** | **Post hoc tests** | **Main effect of group** |
| --- | --- | --- | --- | --- | --- | --- | --- |
|  | **FEP** | **HC** | **FEP** | **HC** |  |  |  |
| **Thalamus rCBF**  in mL/100g/min ± SD | 60.3±14.6 | 61.5±15.0 | 61.1±14.8 | 56.9±14.7 | p=0.026 | FEP = HC baseline: p=0.96  FEP > HC 6 weeks: p=0.05 | - |
| **Whole striatum**  in mL/100g/min ± SD | 41.3±7.8 | 41.8±9.4 | 43.1±8.5 | 40.1±6.9 | P=0.046 | FEP = HC baseline: p=0.85  FEP > HC 6 weeks: p=0.04 |  |
| **Putamen rCBF**  in mL/100g/min ± SD | 42.4±8.8 | 43.8±10.9 | 45.0±8.9 | 41.6±7.7 | p=0.012 | FEP = HC baseline: p=0.53  FEP > HC 6 weeks: p=0.027 | - |
| **Nucleus accumbens rCBF**  in mL/100g/min ± SD | 35.6±9.7 | 37.5±12.3 | 40.0±10.2 | 35.7±8.8 | p=0.004 | FEP = HC baseline: p=0.38  FEP > HC 6 weeks: p=0.025 | - |
| **Caudate rCBF**  in mL/100g/min ± SD | 43.9±9.6 | 43.6±9.9 | 45.4±10.5 | 42.8±8.3 | p=0.26 | - | p=0.38 |
| **Hippocampus rCBF**  in mL/100g/min ± SD | 30.7±7.5 | 32.1±9.8 | 31.6±7.5 | 29.8±6.5 | p=0.05 | FEP = HC baseline: p=0.67  FEP > HC 6 weeks: p=0.10 | - |
| **Frontal lobe rCBF**  in mL/100g/min ± SD | 56.7±57.2 | 57.2±11.4 | 55.7±9.9 | 54.8±10.4 | p=0.45 | - | p=0.79 |
| **Global rCBF**  in mL/100g/min ± SD | 50.7±8.4 | 51.4±9.3 | 49.6±8.1 | 48.7±8.3 | p=0.34 | - | p=0.75 |

Supplementary Table S2 shows regional cerebral blood flow in regions of interest in initially antipsychotic-naïve patients with schizophrenia or psychosis and matched healthy controls in analyses not adjusted for age, sex, smoking status, and global rCBF.

Abbreviations: FEP: First-episode patients with psychosis or schizophrenia; HC: Healthy controls; rCBF: resting cerebral blood flow; SD; standard deviation.

**References**

1. Alisch JSR, Khattar N, Kim RW, Cortina LE, Rejimon AC, Qian W, et al. Sex and age-related differences in cerebral blood flow investigated using pseudo-continuous arterial spin labeling magnetic resonance imaging. Aging (Albany NY). 2021;13(4):4911-25.

2. Elbejjani M, Auer R, Dolui S, Jacobs DR, Jr., Haight T, Goff DC, Jr., et al. Cigarette smoking and cerebral blood flow in a cohort of middle-aged adults. J Cereb Blood Flow Metab. 2019;39(7):1247-57.
